# Socioeconomic status and its limited influence on perceptions of heated tobacco products and cigarettes: no relation with physical health, but association with mental health benefits and lower sensitivity to peer pressure

**DOI:** 10.3389/fpubh.2025.1586447

**Published:** 2025-05-22

**Authors:** Magdalena Kozela, Janusz Sytnik-Czetwertyński, Maciej Polak, Barbara Gradowicz-Prajsnar, Maciej Rogala

**Affiliations:** ^1^Department of Epidemiology and Population Studies, Jagiellonian University Medical College, Krakow, Poland; ^2^Centre of Postgraduate Medical Education, Warsaw, Poland; ^3^Department of Health Policy and Management, Jagiellonian University Medical College, Krakow, Poland

**Keywords:** socioeconomic status, heated tobacco products, tobacco, smoking, physical wellbeing, mental wellbeing, peer pressure

## Abstract

**Introduction:**

Socioeconomic status is related with individuals’ attitudes toward health behaviors and perceptions of risk. This study investigated the relationships between socioeconomic status and perceptions of the impact of heated tobacco products (HTPs) and cigarette smoking on the physical, mental, and social well-being of users.

**Methods:**

A cross-sectional study was conducted using a population-based random sample of 2,500 HTP users and former smokers over the age of 25. The computer-assisted web interview (CAWI) method was employed to gather data. Information on gender, age, education, place of residence, income, and detailed perceptions of the impact of HTPs use and cigarette smoking on physical, mental, and social well-being was collected. A socioeconomic status score was derived based on education and income data. Multivariable multinomial regression analysis was used to assess the impact of socioeconomic status on perceptions of HTPs use and cigarette smoking in relation to physical, mental, and social well-being, controlling for age, place of residence, and perceived health status. The reference category was middle socioeconomic status and the middle category of perceived impact.

**Results:**

A total of 2,254 participants were included in the analysis. Socioeconomic status was not related with perceptions of the impact of HTPs use or cigarette smoking on physical well-being. Compared to those with middle socioeconomic status, individuals with low socioeconomic status were more likely to perceive a positive impact of HTPs use on mental well-being (OR = 1.71, 95% CI: 1.12–2.60). Women with low socioeconomic status showed a stronger perception of being unaffected by peer pressure, both against smoking cigarettes and using HTPs (OR = 1.69, 95% CI: 1.11–2.57; OR = 1.53, 95% CI: 1.10–2.12, respectively).

**Conclusion:**

While socioeconomic status did not differentiate perceptions of the impact of HTPs use or smoking on physical health, more tailored public health strategies that consider socioeconomic factors may be needed when addressing mental health perceptions and the influence of peer pressure.

## 1 Introduction

Tobacco use is the leading preventable cause of death both in Europe and globally. Although the harmful effects of tobacco smoking have been well-established through reliable research for at least 70 years, population-based efforts to reduce smoking remain insufficient (1–3). The World Health Organization encourages the goal of becoming tobacco-free populations, where smoking prevalence does not exceed 5%. While a decline in cigarette smoking prevalence has been observed in Europe, the rate of decline is far too slow to achieve the target by 2030 (4). In Poland, it is estimated that over 20% of adults are regular smokers (5, 6). In Western European countries, the rate is slightly lower, but the most favorable rates are found in the Nordic countries. For example, in Sweden, the prevalence of tobacco smoking has fallen below 10% (6). As in other countries, cigarette smoking is strongly inversely related to socioeconomic status.

Heated tobacco products (HTPs), introduced in recent years, are designed to heat tobacco to a temperature high enough to release vapor without burning it and producing smoke. HTPs likely expose users to fewer toxins than cigarettes, but possibly more than not using any tobacco at all (7). A systematic review of the adverse effects of HTP use indicated that HTPs may be considered products with a reduced risk of chronic diseases for smokers, but they may increase the risk of these diseases in non-smokers (8). In July 2020, the U.S. Food and Drug Administration (FDA) granted limited authorization to market IQOS (an HTP produced by Philip Morris International) as a modified-risk tobacco product, allowing claims that IQOS reduces exposure to harmful chemicals, but not allowing claims that it reduces harm (9). Following the launch of HTPs in Japan, cigarette sales declined more rapidly, although it is uncertain whether this can be attributed to a switch from cigarettes to heated tobacco. Comparisons across countries suggest that nations with higher adoption rates of alternative nicotine products have achieved lower smoking rates. These findings suggest that the introduction of alternative nicotine products may help reduce smoking prevalence more quickly than focusing solely on prevention and smoking cessation (10). However, the results of the Cochrane review on the use of HTPs for smoking cessation and reducing smoking prevalence highlighted the limited reliability of analyses based on trend comparisons only (7).

In some countries, the use of HTPs has become very popular, reaching 11% of the total tobacco market in South Korea in 2020, and also in Japan (11, 12). Studies conducted in these populations revealed that the most common reasons for initiating HTPs use among all consumers were: curiosity (58.9%), family and friends using HTPs (45.5%), and an interest in the technology behind HTPs (35.9%). Regular use of HTPs was most often driven by the fact that they were less smelly than cigarettes (71.3%), beliefs that HTPs are less harmful to health than cigarettes (48.6%), and the perceived stress-reducing effects of HTPs (47.4%). Overall, about one-third of HTPs consumers reported using these devices to quit smoking, 14.7% used them to reduce smoking but not to quit, while half of all consumers (49.7%) used HTPs for other reasons, suggesting that the majority of HTPs users in South Korea had no intention of using them as an aid to quit smoking. In a Japanese study, the most common reasons for regular HTPs use were beliefs that HTPs are less harmful than cigarettes (90.6%), enjoyment (76.5%), and social acceptability (74.4%). Over half of smokers reported using HTPs as an aid to quit smoking. However, the other half used HTPs to replace some cigarettes, meaning they did not intend to quit smoking entirely. With this approach, the risk-reduction potential of HTPs, as suggested by toxicity studies, may be substantially diminished. Data from Europe show that, in 2017–2018, HTPs use remained limited in the general population. However, the dual use of these products alongside cigarettes, their high use among younger generations, and the interest in these products from non-smokers are concerning, as they may indicate a growing public health issue (13).

Data from HTPs users in Canada, England, the United States, and Australia indicated that cigarette smokers who used HTPs appeared more interested in quitting. Both the intention to quit smoking within 6 months and a history of failed quit attempts were positively associated with current HTPs use. It was reported that, compared to non-users, current HTP users were younger and had higher socioeconomic status (14). A Chinese study also confirmed a positive association between socioeconomic status and HTPs use, as well as the intention to use HTPs (15). Similarly, in South Korea, a positive association was found between socioeconomic status and subsequent HTPs use among ever-smokers (16). HTPs users were more likely than non-users to perceive HTPs as less harmful than cigarettes, and the stronger this perception, the more frequently HTPs were used. Smokers who had been exposed to HTPs advertising were more likely to perceive HTPs as less harmful than cigarettes (17). Socioeconomic status is not only associated with smoking behaviors but may also shape perceptions toward the health impacts of tobacco products. In Japanese study tobacco users were more likely to perceive HTPs as less harmful compared to non-users, but younger age and low education both among users and non-users were related to perception of lower harmfulness of HTPs compared to traditional cigarettes. The mechanisms linking socioeconomic status to perceptions of the health effects of HTPs use may involve several mechanisms, including variations in risk perception, health literacy, as well as differences in chronic stress or coping strategies across different social strata (18–21).

The primary aim of this study was to examine the relationship between socioeconomic status and perceptions of the impact of HTPs use and cigarette smoking on users’ physical, mental, and social well-being.

## 2 Materials and methods

A cross-sectional study was conducted using a random population sample. Collaboration was established with the Public Opinion Research Center (Centrum Badania Opinii Społecznej - CBOS) as the leading partner. CBOS is a publicly funded, independent research center, one of the largest and most renowned public opinion research institutes in Poland. Through CBOS, direct research contractors were engaged: the IQS Think Forward Research Institute and Pollster. Each contractor recruited study participants from their respective representative panels. Participants who met the following inclusion criteria were included: Polish citizenship, over 18 years of age, smoking cigarettes for at least 1 year in the past, and then - after quitting smoking use HTP only, for at least 6 months. These conditions were designed to ensure that the study sample represented individuals who currently use HTP but have ceased cigarette smoking. The study utilized the computer-assisted web interview (CAWI) method, with groups independently recruited by each contractor. The research was conducted simultaneously by both contractors, who adhered strictly to the same standardized research protocol, with the aim of examining at least 1,250 individuals.

The final study group consisted of 2,500 participants. The interview collected data on gender, age, education, place of residence, and income. Detailed self-reported information was gathered on the perceived impact of cigarette smoking or HTP use on fitness (endurance), mental health and perceived peer pressure against smoking cigarettes or using HTPs. Since the participant structure across the two research contractors was consistent, the data were combined, and the analysis was conducted on the entire sample.

Socioeconomic status was defined using the method developed by Kozakiewicz et al. in the WOBASZ Study, based on the experience from the ATTICA Study (22, 23). The socioeconomic status score was calculated by multiplying ordinal numerical values assigned to consecutive categories of education and income level. Education categories were as follows: primary = 1, vocational = 2, secondary = 3, bachelor’s degree = 4, and master’s degree or PhD = 5. Income in PLN was categorized as: <3,000 = 1, 3,000–4,999 = 2, 5,000–9,999 = 3, and ≥10,000 = 4. Responses indicating “I am supported by others,” which accounted for approximately 4% of all responses, were excluded. The socioeconomic status score ranged from 1 to 20. For further analysis, participants were divided into three subgroups based on tertile distribution: low (0–5), medium (6–9), and high (7, 10–19) socioeconomic status. Given that the socioeconomic status index score was determined based on income and education, participants under the age of 25 could not achieve the highest possible score solely due to their age, as the completion of a Master’s degree in Poland typically occurs at age 24. Inclusion of younger participants would result in a systematic decrease in the SES index, which would be attributable solely to age. To mitigate this possible bias, we decided to include only participants who were able to have reached their highest level of education by the age of 25.

Continuous variables were presented as medians with first and third quartiles (Q1-Q3). Categorical variables were reported as counts and percentages. Multivariable multinomial regression analysis was conducted, adjusting for age, place of residence, and perceived health status. The reference category was middle socioeconomic status (SES) and the middle category of perceived impact. The results were expressed as odds ratios (OR) with 95% confidence intervals (CI) and *p*-values. Given that men and women differ in the distributions of basic characteristics and that cultural gender differences may also play a role and the presence of significant interaction terms between the gender and socioeconomic status for some outcomes, gender-specific analyses were conducted. Results of combined analysis are also available in Supplementary Table 1. The analysis was done using IBM SPSS Statistics for Windows, Version 28.0 (IBM Corp., Armonk, NY, 2021) or R version 4.0.5 (R Core Team, 2021, R Foundation for Statistical Computing, Vienna, Austria). *p*-values < 0.05 were considered statistically significant.

## 3 Results

**Figure 1:**
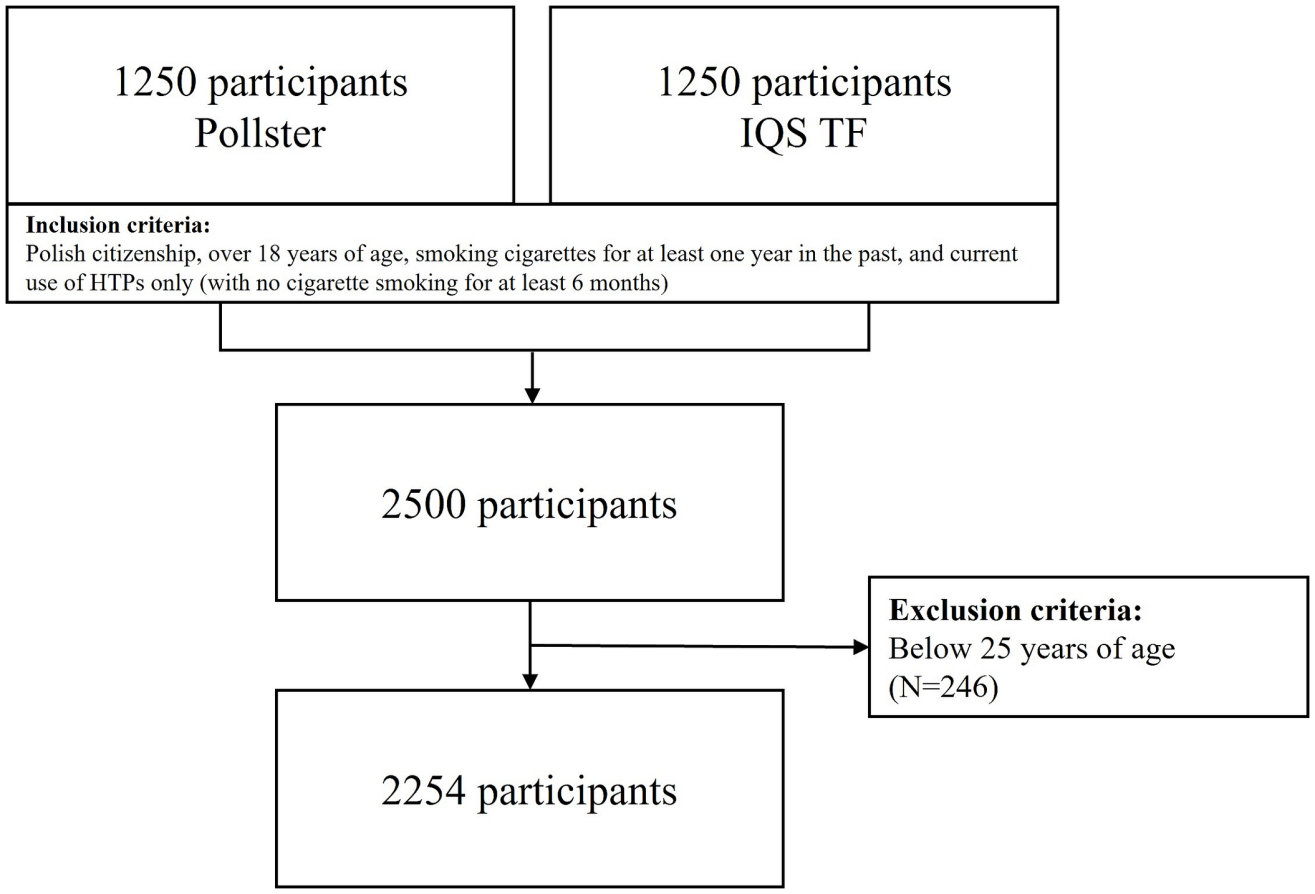
Study participants.

A total of 2,254 participants (62% women) were included in the analysis (Figure 1). The median age in women was 35.5 years (Q1 = 30, Q3 = 44) and in men 40 years (Q1 = 33, Q3 = 49). In total sample 65% of participants had a university education (bachelor’s degree or higher), but compared to men, higher proportion of women had university education (68% vs. 60%, respectively). Approximately half of the participants reported a monthly income between 3,000 and 4,999 PLN, but on average men had higher income and higher SES. About 15% of women and 12% of men declared living in rural areas, while the majority of respondents resided in small and medium-sized towns. Women assessed their health condition worse than men (24.3% vs. 33.6% of participants with very good or good perceived health, respectively). The most frequent experiences related to replacing cigarettes with HTPs were: feeling of increased comfort of life (27%) and motivation for major lifestyle changes (25%) in women while in men motivation for major lifestyle changes (28%) was followed by mobilization to decide to quit the addiction (23%). Regardless of gender, almost half of the participants stated they were well-informed about the harmful effects of cigarettes and HTPs. However, 15% of women and 14% of men admitted they were not informed about the harmfulness of smoking or using HTPs, but did not consider it necessary to be informed. Nearly three-quarters of participants indicated that state-provided information on the harmfulness of cigarettes is easily accessible, but only 36.2% found it sufficient (Table 1).

**Table 1:** Descriptive statistics of studied group.

	Women 1,380 (61.2)	Men 874 (38.8)	*p*-value
	Me	Q1-Q3	
Age [years]	35.5 (30–44)	40 (33–49)	**<0.001**
	n	%	
Education			
Primary	9 (0.6)	14 (1.6)	**0.001**
Vocational	121 (8.8)	93 (10.6)	
Secondary school	316 (22.9)	245 (28.0)	
Bachelor degree	378 (27.4)	206 (23.6)	
Master or PhD	556 (40.3)	316 (36.2)	
Monthly income			
0–2,999 PLN	402 (29.1)	126 (14.4)	**<0.001**
3,000–4,999 PLN	684 (49.6)	423 (48.4)	
5,000–9,999 PLN	241 (17.5)	255 (29.2)	
≥10,000 PLN	53 (3.8)	70 (8.0)	
Socioeconomic status			
Low	449 (32.6)	175 (20.0)	**<0.001**
Middle	391 (28.3)	329 (37.7)	
High	540 (39.1)	370 (42.3)	
Place of residence			
Countryside	213 (15.4)	105 (12.0)	**<0.01**
City less than 50,000 inhabitants	421 (30.5)	316 (36.2)	
City 50.000–1 00.0000 inhabitants	447 (32.4)	260 (29.7)	
City 500.000 inhabitants or more	299 (21.7)	193 (22.1)	
Perceived health			
Very good or good	335 (24.3)	294 (33.6)	**<0.001**
Moderate or bad	1,045 (75.7)	580 (66.4)	
Experiences related to replacing cigarettes with HTP			
Mobilization to decide to quit the addiction	266 (19.3)	199 (22.8)	**0.001**
Motivation for major lifestyle changes	351 (25.4)	244 (27.9)	
Last resort to quit smoking	188 (13.6)	141 (16.1)	
Feeling of increased comfort of life	375 (27.2)	178 (20.4)	
No change	200 (14.5)	112 (12.8)	
Being well informed about the harmfulness of cigarettes and HTP			
No. not considered necessary	210 (15.2)	122 (13.9)	0.44
No. do not know where to get information from	265 (19.2)	150 (17.2)	
Yes. Knows everything considered necessary	649 (47.0)	429 (49.1)	
Yes. But there is a need for additional support	256 (18.6)	173 (19.8)	
State information on the of harmfulness of cigarettes			
Easily accessible and sufficient	500 (36.2)	315 (36.0)	0.62
Easily accessible but insufficient	516 (37.4)	325 (37.2)	
Hardly accessible. But sufficient	165 (12.0)	119 (13.6)	
Hardly accessible and insufficient	199 (14.4)	115 (13.2)	
Perceived impact of smoking cigarettes on fitness (endurance)			
Good	146 (10.6)	109 (12.5)	**0.03**
No	380 (27.5)	199 (22.8)	
Bad	854 (61.9)	566 (64.7)	
Perceived impact of HTP use on fitness (endurance)			
Good	326 (23.6)	191 (21.9)	0.42
No	655 (47.5)	410 (46.9)	
Bad	399 (28.9)	273 (31.2)	
Perceived impact of HTP use on mental condition			
Good	397 (28.8)	245 (28.0)	0.36
No	829 (60.1)	514 (58.8)	
Bad	154 (11.1)	115 (13.2)	
Thoughts about returning to smoking cigarettes			
No	883 (64.0)	532 (60.9)	**0.03**
Do not know	320 (23.2)	245 (28.0)	
Yes	177 (12.8)	97 (11.1)	
Perceived peer pressure against smoking cigarettes			
Often	777 (56.3)	484 (55.4)	0.47
Rarely	398 (28.8)	271 (31.0)	
Never	205 (14.9)	119 (13.6)	
Perceived peer pressure against using HTP			
Often	297 (21.5)	180 (20.6)	0.35
Rarely	730 (52.9)	489 (55.9)	
Never	353 (25.6)	205 (23.5)	
Influence of peer pressure against smoking cigarettes			
Great	810 (58.7)	532 (60.9)	0.20
Little	298 (21.6)	196 (22.4)	
No	272 (19.7)	146 (16.7)	
Influence of peer pressure against using HTP			
Great	438 (31.7)	319 (36.5)	**0.007**
Little	477 (34.6)	312 (35.7)	
No	465 (33.7)	243 (27.8)	

HTP, heated tobacco products; PLN, Polish zloty; Significant results in bold.

Table 2 presents the adjusted associations between socioeconomic status and the perceived impact of HTPs use or cigarette smoking on physical, mental, and social well-being. The perceived impact of HTPs use or cigarette smoking on fitness (endurance) was independent of the users’ socioeconomic status. Socioeconomic status also did not differentiate the perception of the impact of cigarette smoking on mental health in women. However, compared to men with middle socioeconomic status, men with low socioeconomic status were 71% more likely to report a positive impact of HTPs use on mental health (OR = 1.71, 95% CI = 1.12–2.6). Women with low socioeconomic status were more likely to disregard peer pressure against smoking (OR = 1.95, 95% CI = 1.26–3.04) or HTPs use (OR = 1.54, 95% CI = 1.11–2.15) than women with medium socioeconomic status. Additionally, low socioeconomic status in women was associated with the perception of being unaffected by peer pressure against both smoking cigarettes and using HTPs (OR = 1.69, 95% CI = 1.11–2.57; OR = 1.53, 95% CI = 1.10–2.12, respectively).

**Table 2:** The associations between socioeconomic status and perceived impact of smoking or HTP use on physical, mental well-being and perceived peer pressure in men and women.

Socioeconomic status	Women					Men				
	OR^a^ (95%CI)	*p*-value		OR^a^ (95%CI)	*p*-value	OR^a^ (95%CI)	*p*-value		OR^a^ (95%CI)	*p*-value
	Perceived impact of smoking cigarettes on fitness (endurance)					Perceived impact of smoking cigarettes on fitness (endurance)				
	Good		No	Bad		Good		No	Bad	
Low	1.33 (0.8–2.21)	0.279	Ref.	0.93 (0.68–1.27)	0.631	1.81 (0.95–3.45)	0.072	Ref.	0.7 (0.44–1.12)	0.138
Mod	Ref.		Ref.	Ref.		Ref.		Ref.	Ref.	
High	1.57 (0.96–2.59)	0.075	Ref.	1.08 (0.8–1.46)	0.624	1.21 (0.69–2.13)	0.511	ref.	0.73 (0.5–1.06)	0.097
	Perceived impact of HTP use on fitness (endurance)					Perceived impact of HTP use on fitness (endurance)				
	Perceived impact of smoking cigarettes on mental condition					Perceived impact of smoking cigarettes on mental condition				
	Good		No	Bad		Good		No	Bad	
Low	0.91 (0.61–1.34)	0.616	ref.	1.06 (0.76–1.46)	0.744	1.24 (0.74–2.06)	0.416	Ref.	0.89 (0.58–1.36)	0.591
Mod	Ref.	Ref.	Ref.	Ref.	Ref.	Ref.	Ref.	Ref.	Ref.	
High	1.1 (0.76–1.58)	0.607	ref.	0.79 (0.58–1.08)	0.146	1.31 (0.85–2.02)	0.224	Ref.	1.25 (0.87–1.77)	0.224
	Perceived impact of HTP use on mental condition					Perceived impact of HTP use on mental condition				
	Good		No	Bad		Good		No	Bad	
Low	0.84 (0.62–1.15)	0.289	Ref.	1.27 (0.8–2.01)	0.311	**1.71 (1.12–2.6)**	**0.012**	Ref.	1.22 (0.69–2.17)	0.492
Mod	Ref.		Ref.	Ref.		Ref.		Ref.	Ref.	
High	1.01 (0.76–1.36)	0.924	Ref.	1.26 (0.8–1.98)	0.324	1.01 (0.71–1.45)	0.939	Ref.	1.14 (0.72–1.83)	0.577
	Thoughts about returning to smoking cigarettes					Thoughts about returning to smoking cigarettes				
	No		Do not know	Yes		No		Do not know	Yes	
Low	0.92 (0.66–1.29)	0.624	Ref.	1.14 (0.72–1.81)	0.589	1.08 (0.7–1.66)	0.723	Ref.	0.54 (0.26–1.12)	0.098
Mod	Ref.		Ref.	Ref.		Ref.		Ref.	Ref.	
High	0.98 (0.71–1.36)	0.916	Ref.	0.74 (0.46–1.19)	0.211	0.95 (0.67–1.35)	0.78	Ref.	0.84 (0.5–1.42)	0.506
	Perceived peer pressure against smoking cigarettes					Perceived peer pressure against smoking cigarettes				
	Often		Rarely	Never		Often		Rarely	Never	
Low	0.92 (0.67–1.27)	0.622	Ref.	**1.95 (1.26–3.04)**	**0.003**	0.73 (0.48–1.12)	0.154	Ref.	1.07 (0.59–1.93)	0.83
Mod	Ref.		Ref.	Ref.		Ref.		Ref.	Ref.	
High	1.01 (0.75–1.37)	0.932	Ref.	0.98 (0.62–1.55)	0.943	**0.62 (0.44–0.87)**	**0.007**	Ref.	0.67 (0.4–1.11)	0.118
	Perceived peer pressure against using HTP					Perceived peer pressure against using HTP				
	Often		Rarely	Never		Often		Rarely	Never	
Low	1.16 (0.81–1.66)	0.409	Ref.	**1.54 (1.11–2.15)**	**0.01**	0.8 (0.48–1.33)	0.386	Ref.	1.13 (0.72–1.78)	0.583
Mod	Ref.		Ref.	Ref.		Ref.		Ref.	Ref.	
High	1.24 (0.89–1.74)	0.205	Ref.	1.03 (0.75–1.43)	0.845	1.02 (0.69–1.51)	0.915	Ref.	0.94 (0.64–1.37)	0.736
	Influence of peer pressure against smoking cigarettes					Influence of peer pressure against smoking cigarettes				
	Great		Little	No		Great		Little	No	
Low	0.97 (0.69–1.37)	0.87	Ref.	**1.69 (1.11–2.57)**	**0.015**	1.39 (0.86–2.24)	0.178	Ref.	1.19 (0.63–2.23)	0.589
Mod	Ref.		Ref.	Ref.		Ref.		Ref.	Ref.	
High	1.28 (0.92–1.77)	0.142	Ref.	1.04 (0.68–1.6)	0.86	0.87 (0.6–1.26)	0.449	Ref.	1.05 (0.65–1.71)	0.835
	Influence of peer pressure against using HTP					Influence of peer pressure against using HTP				
	Great		Little	No		Great		Little	No	
Low	1.29 (0.91–1.83)	0.159	Ref.	**1.53 (1.1–2.12)**	**0.011**	1.03 (0.66–1.61)	0.889	Ref.	1.15 (0.72–1.82)	0.568
Mod	Ref.		Ref.	Ref.		Ref.		Ref.	Ref.	
High	**1.48 (1.07–2.05)**	**0.017**	Ref.	0.86 (0.62–1.18)	0.347	1.38 (0.96–1.99)	0.081	ref.	1.44 (0.98–2.13)	0.065

^a^Adjusted for age, place of residence, and perceived health status; Significant results in bold.

## 4 Discussion

Our results suggest that socioeconomic status does not differentiate the perception of the impact of cigarette smoking or HTPs use on physical well-being. This may be due to the widespread knowledge of the harmful effects of these substances, which appears to be similarly distributed across the population. As a result, no differences were observed based on socioeconomic status. However, low socioeconomic status was associated with the perception of a beneficial impact of HTPs use on mental well-being in men. This finding may reflect some cultural gender-specific factors that play a role in shaping men’s perceptions of tobacco use, including newer tobacco alternatives. In women with low socioeconomic status, a strong independence from peer pressure against both cigarette smoking and HTP use was observed. This may reflect an internalized awareness of the harmful effects of tobacco use on their health and well-being.

In Korean studies among cigarette smokers, approximately half of the participants perceived both HTPs and nicotine vaping products as equally harmful as cigarettes. Over 25% of respondents considered HTPs less harmful than cigarettes, while nearly 8% viewed HTPs as more harmful than cigarettes (24). HTPs users tended to assess HTPs more favorably in terms of smoke, smell, harm, aid in quitting, design, and price compared to users of other products (25). American data indicated that about 50% of both ever and current HTPs users considered HTPs less harmful than cigarettes, and over 50% stated that HTPs are socially acceptable (26). Explanatory studies suggest that the perception of HTPs may largely depend on cultural factors. Positive evaluations of HTPs may be stronger in cultures that value purity, exclusivity, and technologically advanced aesthetics. In communities where cigarette smoking is seen as an expression of freedom and individualism, the value of HTPs may be perceived as lower (27). Additionally, this perception may vary within a single community, influenced by differences in socioeconomic status.

However, the majority of quantitative evidence on the perceived impact of cigarette smoking or HTPs use comes from high-income countries and does not explore further socioeconomic differences. Data from the United Kingdom provide deeper insight into the socioeconomic disparities associated with the use of alternative smoking products. A qualitative study of current and former HTPs users in London identified six key factors influencing the initiation and use of HTPs. In addition to health-related factors and the expected harm reduction or long-term financial benefits, sensory experiences such as discretion, cleanliness, reduced odor, and the practical benefits of accessibility in smoke-free environments were noted. Psychological factors, such as the similarity to smoking rituals and routines, as well as enhanced social interactions from using HTPs, were also identified (28). A cross-sectional study on e-cigarette use among former smokers in England found an overall increase in e-cigarette use among individuals who had not smoked for at least 1 year. However, the highest increase was observed among participants with low socioeconomic status (29). Additionally, the UK Household Longitudinal Study demonstrated that socioeconomic disadvantage was associated with e-cigarette use among ex-smokers (OR: 1.17; 95% CI: 1.09–1.26) (30). Moreover, Four Country Survey (ITC-4) showed that lower levels of education were associated with higher nicotine dependence across countries. Respondents with lower education had lower self-efficacy and were more likely to have no intention of quitting compared to those with higher income (31).

Our result of a positive impact of HTP use on mental well-being among male participants with low socioeconomic status is intriguing. Although the possibility that this finding may be attributable to random variation cannot be entirely ruled out, a review of the existing literature suggests notable gender differences in this regard. Cultural and gender-specific factors play a critical role in shaping men’s perceptions of tobacco use, influencing both their attitudes toward traditional tobacco products and newer alternatives. Research has shown that gender norms can affect how men engage with tobacco use and it is associated with masculinity in many cultures. Scoping review by Kodriati et al. revealed that men often associated their smoking behavior with perceptions of being powerful, being emotionally stable, being in control, and having self-reliance. This cultural context and the fact that HTPs are often presented as a “healthier” alternative to traditional cigarettes may influence men’s attitudes toward tobacco use, shaping their perception of its potential mental health benefits (32). Also, men are more likely to use substances like tobacco to cope with stress and negative emotions (33). As a result, tobacco use, including HTPs, may be perceived by men as a means of stress relief or improvement of mental well-being, particularly for those in lower socioeconomic status groups who may face greater stressors.

Public health communications that emphasize the potential negative psychological effects of both cigarette smoking and HTPs use, including mental health distress and the risk of addiction, could play a crucial role in reshaping these perceptions. It is particularly important to highlight the risks associated with HTPs use not only for physical health but also for mental well-being, especially within lower socioeconomic groups, as these individuals appear to underestimate or overlook such threats.

Population studies have identified peer pressure as a key factor influencing smoking behavior patterns. It has been found that individuals with a partner who objects to smoking, those who experience peer pressure to quit, or people living in smoke-free homes are more likely to attempt to quit smoking (34–38). Conversely, higher social acceptance has been observed regarding HTPs use, and interestingly, a substantial proportion of users acquired their devices as gifts from relatives or friends (39). It is also known that gender plays a role in susceptibility to peer pressure, with slightly more boys than girls being vulnerable to peer pressure (40). Our finding of women’s independence from peer pressure against smoking or HTPs use aligns with the results of a study by Tsai et al., which suggested that social peer pressure is more influential on smoking behaviors in men, whereas women are more likely to use smoking as a coping mechanism for psychological distress (41). While available evidence does not fully explain the relationship, it raises questions about the causes of differences in perceptions of peer pressure against smoking or HTPs use, especially by socioeconomic status. In the case of HTPs use in Polish society, it seems plausible that individuals higher in the social hierarchy may be more susceptible to peer pressure.

There are several limitations in interpreting the results that should be considered. First, the study assessed respondents’ perceptions of their feelings, rather than objective measures of their physical and mental health or social functioning. Second, the study group likely consisted of healthier individuals with a higher-than-average socioeconomic status, which may have led to an underestimation of the relationships examined. However, this profile is representative of HTPs users in the Polish population, so the findings can be generalized to this group. To facilitate a comparison between HTPs use and cigarette smoking, former smokers who were current HTP users were included in the study. This may have influenced their perception of cigarettes, potentially leading them to assess cigarettes more negatively and HTP use more favorably, although this effect likely applies uniformly across the entire study group.

Despite the limitations, there are several notable strengths that should be highlighted. This is the first large-scale survey on HTP use conducted in Central and Eastern Europe, a region still facing a slow decline in the prevalence of cigarette smoking. The study uniquely addressed socioeconomic differences in the perception of HTPs and cigarette smoking, offering new insights into this area of research. A large, representative sample of people who use HTP but do not smoke cigarettes was drawn from two independent polling stations, ensuring a similar distribution of sociodemographic characteristics among respondents. Standard research methods were employed, and a well-defined protocol was followed to minimize systematic errors. Associations were assessed after adjusting for potential confounders.

## 5 Conclusion

Low socioeconomic status is related with perceived positive impact of HTP use on the mental well-being of male users, independent of age, place of residence, and self-rated health. Women from lower socioeconomic backgrounds may exhibit greater resistance to peer pressure regarding tobacco use. The unique findings related to psychological well-being in men and resilience to peer pressure in women provide a foundation for more targeted research and interventions. The observed differences in mental health perceptions and sensitivity to peer pressure suggest that tailored messages are needed to address the diverse ways individuals perceive the impact of smoking alternatives like HTPs, as well as to promote healthier coping strategies. Overall, the study findings emphasize the importance of tailoring public health strategies to address the nuanced needs of different socioeconomic groups. While socioeconomic status did not significantly differentiate perceptions of the physical health effects of tobacco, it clearly influences mental health perceptions and the ability to resist peer pressure. Therefore, planned interventions probably should go beyond generic health messaging and include targeted approaches that address both mental health and peer dynamics, particularly for low SES individuals who may be more vulnerable to misperceptions or external social pressures.

## Data Availability

The raw data supporting the conclusions of this article will be made available by the authors, without undue reservation.
