# Supplementary material for: Socioeconomic status and its limited influence on perceptions of heated tobacco products and cigarettes: no relation with physical health, but association with mental health benefits and lower sensitivity to peer pressure
Source: Front Public Health. 2025 May 22;13:1586447. doi: 10.3389/fpubh.2025.1586447 (PMC12137243; doi:10.3389/fpubh.2025.1586447)
Supplement: Supplementary file 1 [file Table_1.docx]

Supplementary Table 1. The associations between socioeconomic status and perceived impact of smoking or HTP use on physical, mental well-being and perceived peer pressure (combined analysis)

| Socioeconomic status | All | | | | | | |
| --- | --- | --- | --- | --- | --- | --- | --- |
|  | OR^a^ (95%CI) | p-value | | |  | OR^a^ (95%CI) | p-value |
|  | Perceived impact of smoking cigarettes on fitness (endurance) | | | | | | |
|  | Good | | | | No | Bad | |
| low | 1.50 (1.01-2.73) | | 0.047 | | ref. | 0.85 (0.65-1.09) | 0.200 |
| mod | ref. | | | | ref. | ref. | |
| high | 1.43 (0.99-2.08-2.56) | | 0.058 | | ref. | 0.93 (0.74-1.18) | 0.555 |
|  | Perceived impact of smoking cigarettes on mental condition | | | | | | |
|  | Good | | | | No | Bad | |
| low | 0.99 (0.75-1.33) | | 0.989 | | ref. | 0.96 (0.74-1.24) | 0.772 |
| mod | ref. | | ref. | | ref. | ref. | ref. |
| high | 1.25 (0.97-1.61) | | 0.090 | | ref. | 1.03 (0.82-1.30) | 0.783 |
|  | Perceived impact of HTP use on mental condition | | | | | | |
|  | Good | | | | No | Bad | |
| low | 1.06 (0.83-1.36) | | 0.643 | | ref. | 1.19 (0.84-1.69) | 0.324 |
| mod | ref. | | | | ref. | ref. | |
| high | 1.02 (0.81-1.28) | | 0.856 | | ref. | 1.17 (0.85-1.62) | 0.333 |
|  | Thoughts about returning to smoking cigarettes | | | | | | |
|  | No | |  | | Don't know | Yes |  |
| low | 0.97 (0.74-1.26) | | 0.81 | | ref. | 0.97 (0.67-1.41) | 0.876 |
| mod | ref. | |  | | ref. | ref. |  |
| high | 0.97 (0.77-1.23) | | 0.808 | | ref. | 0.78 (0.55-1.11) | 0.160 |
|  | Perceived peer pressure against smoking cigarettes | | | | | | |
|  | Often | | | | Rarely | Never | |
| low | 0.84 (0.65-1.09) | | | 0.186 | ref. | 1.56 (1.10-2.21) | 0.012 |
| mod | ref. | | | | ref. | ref. | |
| high | 0.83 (0.66-1.04) | | | 0.103 | ref. | 0.82 (0.59-1.16) | 0.260 |
|  | Perceived peer pressure against using HTP | | | | | | |
|  | Often | | | | Rarely | Never | |
| low | 1.02 (0.77-1.36) | | | 0.880 | ref. | 1.40 (1.07-1.80) | 0.015 |
| mod | ref. | | | | ref. | ref. | |
| high | 1.14 (0.88-1.47) | | | 0.322 | ref. | 0.98 (0.77-1.26) | 0.981 |
|  | Influence of peer pressure against smoking cigarettes | | | | | | |
|  | Great | | |  | Little | No |  |
| low | 1.09 (0.83-1.44) | | | 0.548 | ref. | 1.58 (1.12-2.22) | 0.009 |
| mod | ref. | | |  | ref. | ref. |  |
| high | 1.10 (0.86-1.41) | | | 0.437 | ref. | 1.05 (0.76-1.45) | 0.753 |
|  | Influence of peer pressure against using HTP | | | | | | |
|  | Great | | |  | Little | No |  |
| low | 1.18 (0.90-1.54) | | | 0.242 | ref. | 1.41 (1.08-184) | 0.011 |
| mod | ref. | | |  | ref. | ref. |  |
| high | 1.42 (1.12-1.80) | | | 0.004 | ref. | 1.04 (0.81-1.34) | 0.740 |

a – Adjusted for age, gender, place of residence, and perceived health status
